# Spontaneous Resolution of Miliary Pulmonary Nodules Following Intravesical Bacillus Calmette-Guérin (BCG) Therapy: A Case Report and Literature Review

**DOI:** 10.7759/cureus.88179

**Published:** 2025-07-17

**Authors:** Ahmed R Fadel, Irem G Yaman, Sebastián M Urruela, Sudhir Lohani

**Affiliations:** 1 Respiratory Medicine, Dartford and Gravesham NHS Trust, Dartford, GBR; 2 Microbiology, Dartford and Gravesham NHS Trust, Dartford, GBR; 3 General Internal Medicine, Darent Valley Hospital, Dartford, GBR

**Keywords:** bacillus calmette-guérin (bcg), bacillus calmette-guérin (bcg) immunotherapy, disseminated bcg infection, disseminated mycobacterium bovis infection, hypersensitivity pneumonitis, intravesical therapy complications, miliary pulmonary nodules, non-muscle-invasive bladder cancer

## Abstract

Intravesical Bacillus Calmette-Guérin (BCG) immunotherapy is a well-established treatment for non-muscle-invasive bladder cancer. Although it is typically associated with local irritative symptoms, rare systemic and pulmonary complications can occur, including hypersensitivity pneumonitis and miliary tuberculosis.

We report the case of a 70-year-old man who developed diffuse miliary pulmonary micronodules and ground-glass opacities after his 12th BCG instillation. Despite imaging findings suggestive of disseminated infection, the patient remained clinically stable, without fever, hypoxia, or systemic deterioration. Bronchoalveolar lavage cultures were negative for *Mycobacterium bovis*. A shared decision was made to withhold antimycobacterial therapy and monitor closely. Follow-up imaging revealed spontaneous improvement without treatment.

This case underscores that observation may be appropriate in selected stable patients without systemic signs of infection. Careful clinical assessment and individualized management are essential to avoid unnecessary therapy and ensure patient safety.

## Introduction

Intravesical Bacillus Calmette-Guérin (BCG) immunotherapy is the most effective adjuvant treatment for non-muscle-invasive bladder cancer (NMIBC), especially high-grade Ta/T1 tumors and carcinomas in situ [1]. Its efficacy in reducing recurrence and progression is well established, supporting its use as a first-line therapy for intermediate- and high-risk diseases.

Although local irritative symptoms are common, systemic complications are rare and can be diagnostically challenging. Disseminated BCG infections may present as miliary tuberculosis or granulomatous disease, whereas immune-mediated reactions, including hypersensitivity pneumonitis and eosinophilic pneumonia, can closely mimic infection [2]. Differentiating between these entities is critical as management differs significantly. Radiologic findings, such as diffuse miliary nodules, often raise concerns regarding active infection. However, when microbiological tests are negative and the patient remains clinically stable, a benign hypersensitivity reaction is more likely [3,4].

Here, we describe a patient who developed miliary pulmonary nodules after BCG instillation but remained well without systemic deterioration, ultimately experiencing spontaneous resolution without antimicrobial therapy. This case illustrates that in selected patients without fever, progressive symptoms, or microbiological evidence of infection, close observation may be safe and appropriate.

## Case presentation

A 70-year-old man with a history of high-grade (G3 pT1) urothelial carcinoma of the bladder treated with intravesical BCG immunotherapy presented to the acute medical unit with left-sided pleuritic chest pain and mild shortness of breath. His symptoms began approximately three days after the 12th instillation of BCG. He reported intermittent night sweats lasting 5-6 days but no persistent fever. The patient completed 12 intravesical instillations without prior complications.

His medical history included mild chronic obstructive pulmonary disease (COPD), hypertension, hypercholesterolemia, and type 2 diabetes mellitus. He was an ex-smoker with a 30-pack-year history. He remained independent in activities of daily living and had no recent travel history or known tuberculosis exposure.

On examination, the patient was afebrile with normal oxygen saturation and stable vital signs. Chest auscultation revealed a mild scattered wheeze consistent with his known COPD but no crackles. Lymphadenopathy or hepatosplenomegaly was not observed.

**Figure 1:**
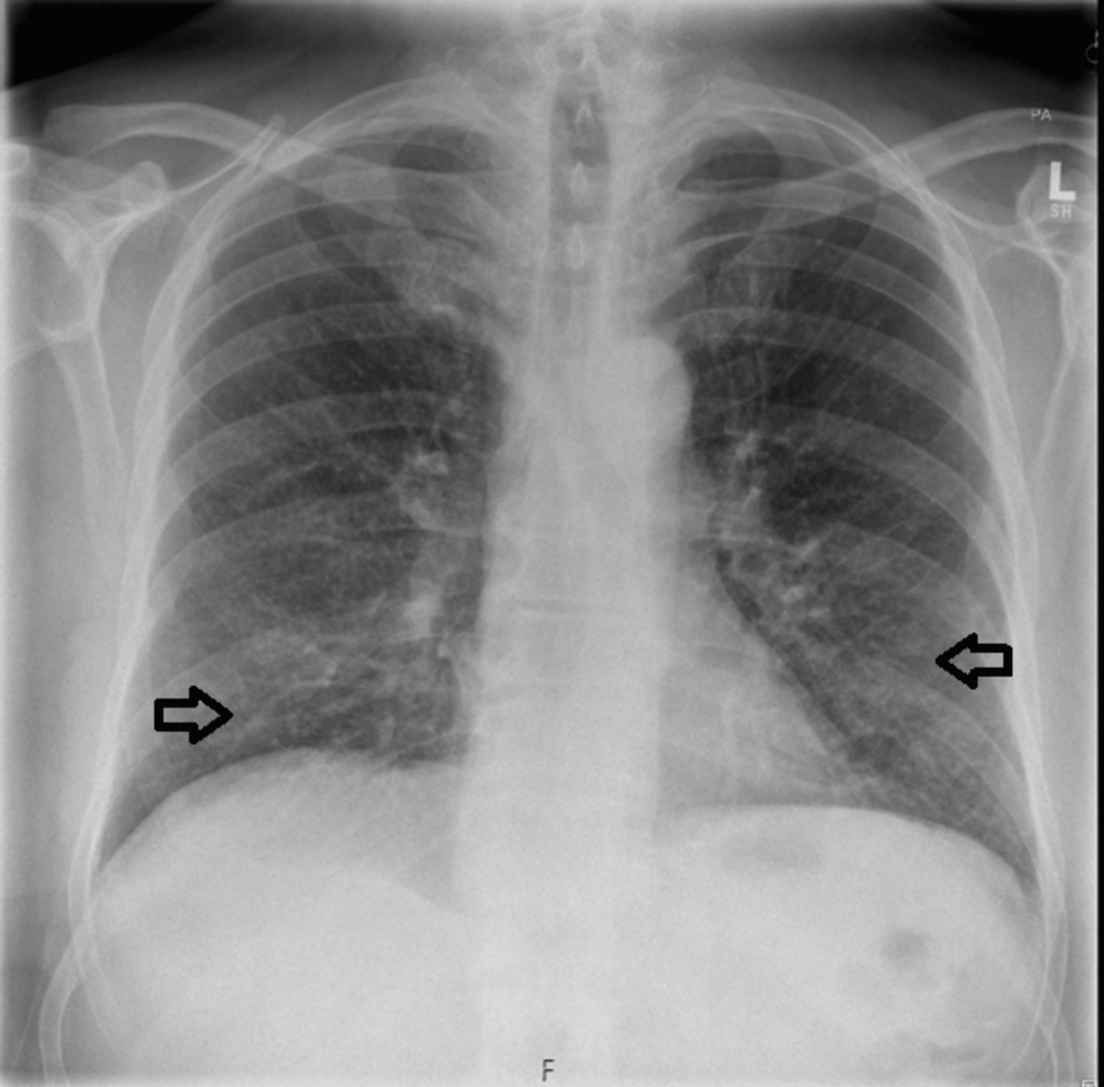
Faint multiple bilateral micronodules

Admission blood tests showed a C-reactive protein (CRP) of 14.4 mg/L (reference range <5 mg/L) and a white cell count of 7.3×10⁹/L (reference range 4-11×10⁹/L). Chest radiography (Figure 1) demonstrated faint multiple bilateral miliary nodules.

**Figure 2:**
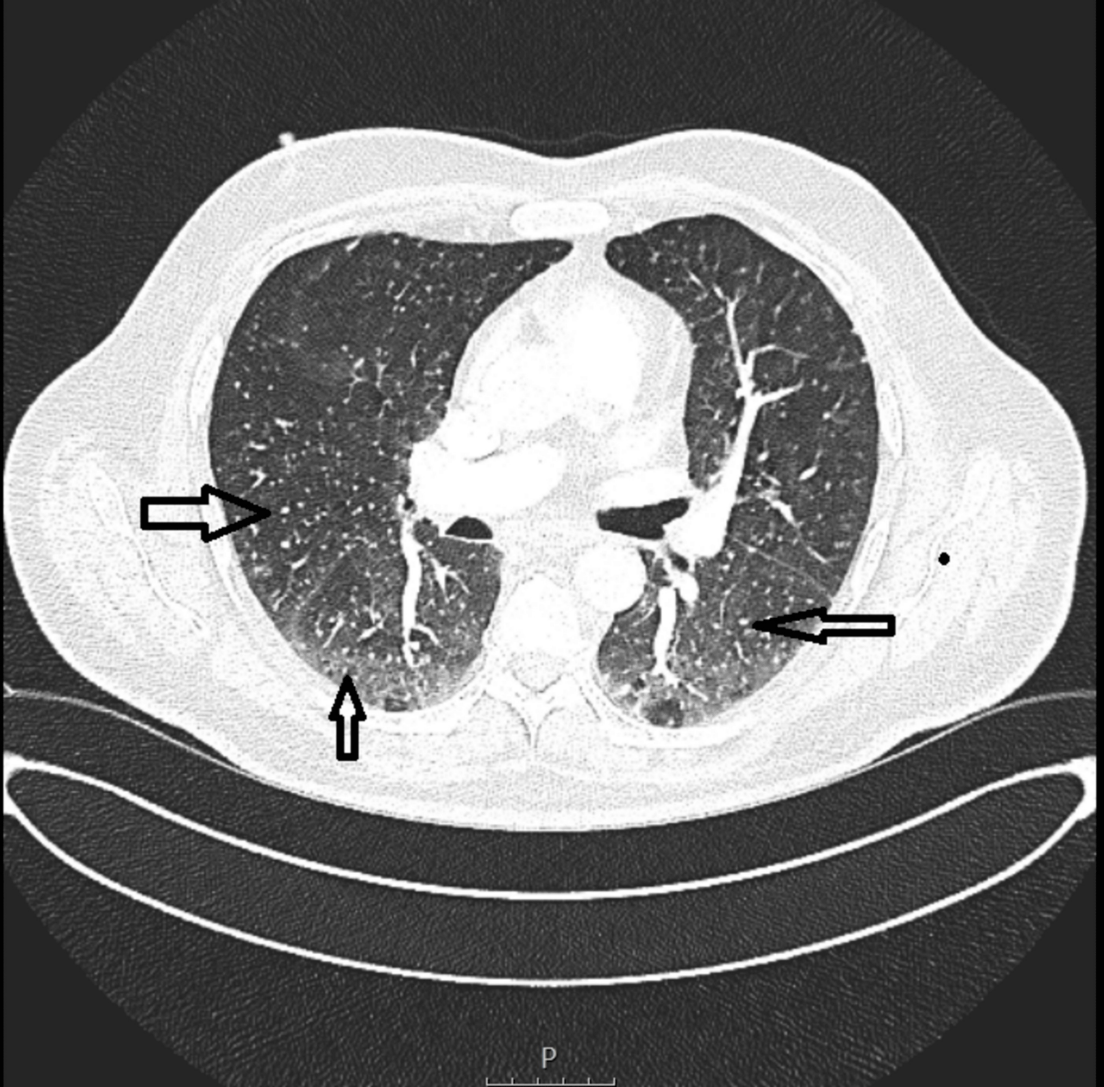
CTPA: Diffuse, uniform miliary nodules measuring 1-3 mm throughout both lungs and patchy bilateral ground-glass opacities (arrows) CTPA: computed tomography pulmonary angiography

To exclude pulmonary embolism due to the pleuritic nature of the pain and to further characterize the pulmonary nodules seen on plain film, computed tomography pulmonary angiography (CTPA) was performed (Figure 2). CTPA excluded pulmonary embolism but demonstrated innumerable bilateral miliary micronodules and patchy bilateral ground-glass opacities. Mediastinal or hilar lymphadenopathy was not observed.

The respiratory team reviewed the patient, who reported a few episodes of shivering but no fever and only occasional mild cough. Given the absence of significant symptoms, immunosuppression, or clinical deterioration, bronchoscopy was performed. Acid-fast bacilli (AFB) cultures of bronchoalveolar lavage (BAL) fluid were negative. BAL cytology revealed abundant benign bronchial epithelial cells with occasional neutrophil polymorphs, alveolar macrophages, benign squamous cells, and scattered lymphocytes. Fungal or malignant cells were not detected.

Transbronchial biopsy was considered but deferred because the patient remained clinically stable and imaging improved spontaneously, favoring a diagnosis of hypersensitivity rather than a disseminated infection. A shared decision was made to avoid initiating antituberculosis treatment. The decision was based on multiple reports supporting individualized management in clinically stable patients without systemic features of infection. The patient was advised to report any new or worsening symptoms urgently, accepted the plan for close follow-up, and was scheduled to return in six weeks.

**Figure 3:**
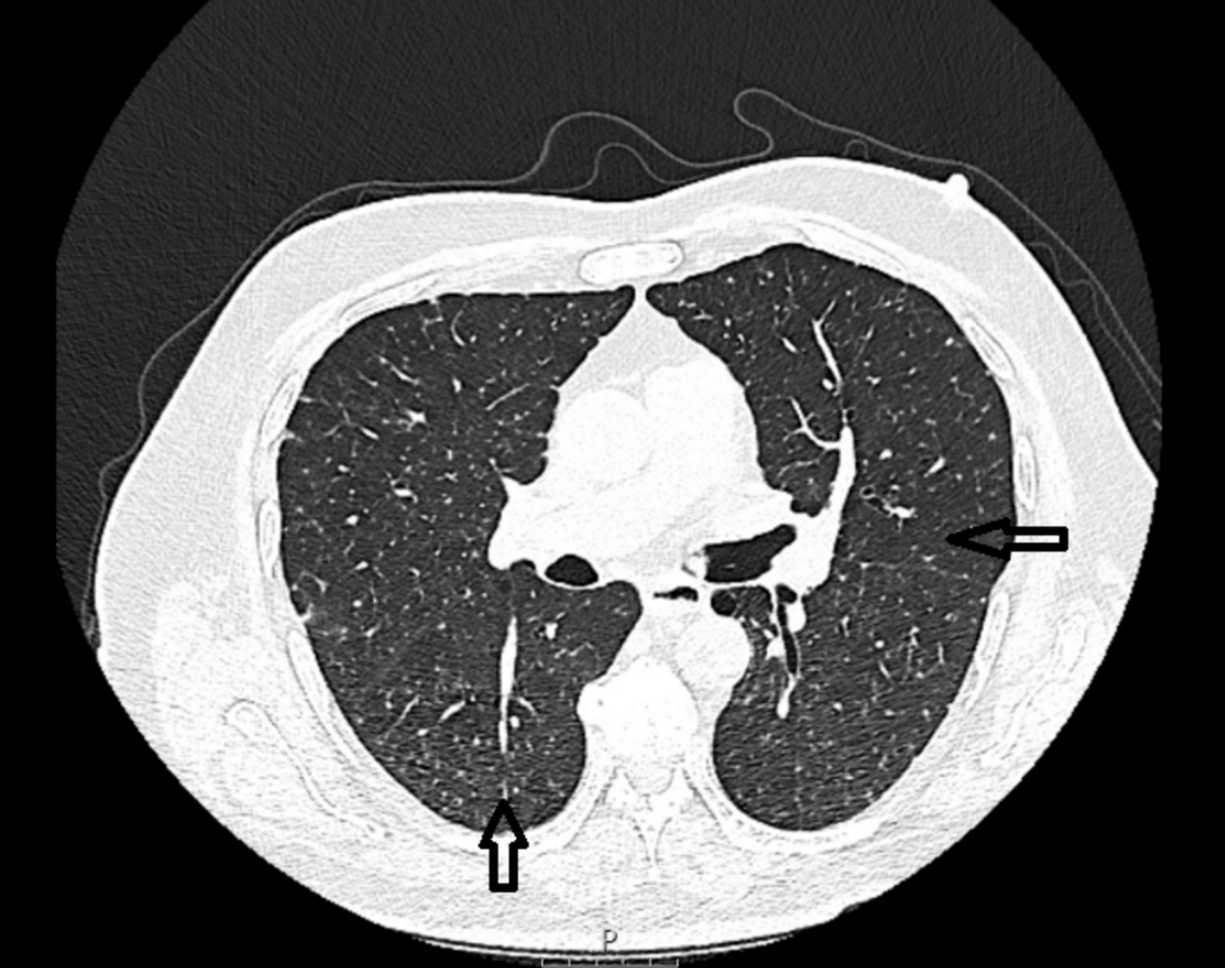
Chest CT: Resolution of the ground-glass opacities and early interval improvement in the miliary micronodules (arrows) CT: computed tomography

At the six-week follow-up appointment, the patient remained asymptomatic, except for a very occasional non-productive cough. He had no fever, weight loss, or other systemic symptoms. High-resolution chest CT demonstrated resolution of the ground-glass opacities and early interval improvement in the miliary micronodules (Figure 3).

On repeat clinical assessment, the patient was afebrile with normal oxygen saturation and an unremarkable chest examination, aside from a mildly scattered wheeze. He remains under close observation, with planned regular follow-up and a strategy to promptly initiate antimycobacterial therapy if any signs or symptoms of actively disseminated BCG infection develop. At the time of submission, the patient remained well without recurrence at the three-month follow-up.

## Discussion

The BCG vaccine was originally developed from a virulent strain of *Mycobacterium bovis* by Calmette and Guérin through serial passaging, resulting in a stable, live attenuated strain used worldwide for tuberculosis prevention. Over time, different laboratories have produced various BCG substrains, such as Pasteur, Danish, Tokyo, and Connaught, which differ in genomic deletions, antigen expression, and immunogenicity [2,5]. These variations have contributed to inconsistent protective efficacy across populations and may also influence the spectrum of adverse reactions observed in clinical practice.

Beyond its role in tuberculosis prevention, BCG remains the most effective intravesical therapy for NMIBC, particularly for high-grade Ta and T1 tumors and carcinoma in situ (CIS). Its antitumor effects are mediated through the activation of both innate and adaptive immune responses within the bladder wall, leading to the cytotoxic elimination of residual cancer cells.

The 2025 European Association of Urology (EAU) guidelines strongly recommend BCG for intermediate- and high-risk NMIBC after complete transurethral resection of bladder tumors (TURBT). Standard induction consists of six weekly instillations followed by maintenance courses to sustain efficacy. Maintenance therapy has been shown to significantly reduce recurrence and progression, with treatment for at least one year advised in high-risk cases and extended regimens considered in selected patients.

Randomized trials have demonstrated that BCG provides superior long-term outcomes compared to intravesical chemotherapy, with complete response rates in CIS approaching 70-80% and durable reductions in recurrence risk for high-grade papillary tumors. Although full-dose BCG is preferred when tolerated, dose reductions may be appropriate during drug shortages or in patients experiencing severe adverse effects.

Although BCG strains vary by region, no single strain has demonstrated clear superiority. During global shortages, guidelines recommend prioritizing high-risk patients and adjusting schedules to maintain outcomes while conserving supplies.

Overall, BCG remains the cornerstone of adjuvant therapy in NMIBC, supported by robust evidence and strong endorsement of guidelines [1].

Adverse events related to intravesical BCG therapy span a wide spectrum, from self-limiting local urinary symptoms to severe systemic complications. In the largest contemporary series by the European Organisation for Research and Treatment of Cancer (EORTC) Genito-Urinary Cancers Group, 69.5% of 1,316 patients experienced adverse effects, most commonly chemical cystitis (35%) and general malaise (15.5%). Approximately 7.8% discontinued treatment due to toxicity [5].

Cystitis remains the most frequent complication, reported in 27-95% of patients across multiple studies, highlighting the need for symptomatic management in nearly all treated individuals [3,6]. Although systemic BCG infection is rare, it can be serious and manifest in diverse ways, including miliary tuberculosis, hepatitis, osteomyelitis, and vascular or ocular involvement. In the EORTC cohort, disseminated infection accounted for 34.4% of systemic events, with other presentations ranging from persistent fever to osteomuscular and pulmonary complications [7].

**Figure 4:**
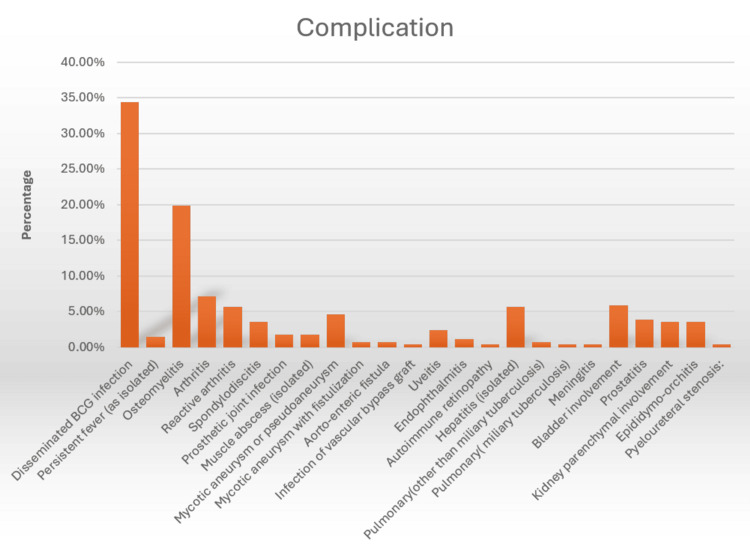
Complications of BCG vaccine instillation Adapted from Pérez-Jacoiste Asín et al. [7] BCG: Bacillus Calmette-Guérin

While pulmonary manifestations are uncommon, clinicians should be aware that miliary nodules, ground-glass opacities, and systemic symptoms can occur, requiring careful distinction between infection and hypersensitivity reactions to guide appropriate management (Figure 4).

BCG-related infections differ from other mycobacterial infections because they are not usually associated with underlying immunodeficiency. In a previous study, only 1.8% of patients had a clear cause of immunosuppression, such as systemic chemotherapy, human immunodeficiency virus (HIV) infection, or prolonged corticosteroid therapy [7].

Instead, mucosal disruption appeared to be the main risk factor for bacterial entry. TURBT is the most common predisposing event reported in nearly all cases. Additional bladder injuries, including photodynamic therapy, fulguration, intravesical chemotherapy, and radiotherapy, were less frequent but also contributed [7].

Comorbidities, such as hypertension, ischemic heart disease, COPD, and peripheral arterial disease, were common in this cohort, reflecting the advanced age and multimorbidity of the patient population. Although it is plausible that these chronic conditions could modestly impair host defenses, a direct causal link to increased risk of BCG infection has not been firmly established [8].

In a study by Pérez-Jacoiste Asín et al. [7], treatment data were available for 274 patients, with 269 (98.2%) receiving therapy. Antituberculosis drugs alone were administered in 46.4% of cases, while combinations of antituberculosis therapy with corticosteroids and surgery were used in 20.1% and 15.7% of cases, respectively. Nonsteroidal anti-inflammatory drugs (NSAIDs) alone were prescribed in 8%, corticosteroids alone in 4.4%, and surgery alone in 2.6% of patients. Other treatment regimens accounted for 1.1%. Only five patients (1.8%) received no specific treatment; notably, one patient with a miliary pattern experienced complete radiological resolution after simply stopping BCG instillations. The most commonly used drug combination included isoniazid, rifampicin, and ethambutol, typically excluding pyrazinamide, with a median treatment duration of six months. A surgical approach, either alone or in combination with medical therapy, was employed in 18.6% of the cases [7,9].

Pulmonary complications after BCG instillation are rare, with an incidence of miliary tuberculosis reported to be 0.4% [1] and non-miliary tuberculosis complications occurring in 0.7% of cases [6]. Non-miliary manifestations may represent hypersensitivity reactions to BCG, resulting in interstitial pneumonitis. Other thoracic manifestations of systemic BCG infection complicating intravesical therapy include focal consolidation or cavitary lung disease indistinguishable from *Mycobacterium tuberculosis* pulmonary infection, empyema, acute eosinophilic pneumonia, and diffuse alveolar damage [10,11].

Hypersensitivity pneumonitis

Hypersensitivity pneumonitis is a rare pulmonary complication of intravesical BCG therapy for bladder cancer and is far less common than miliary tuberculosis. It typically presents with dyspnea, cough, fever, and malaise arising after several instillations, often within days of the third to eighth treatment.

High-resolution CT usually shows diffuse ground-glass opacities and centrilobular nodules, sometimes with lower lobe consolidation. These findings are not specific; therefore, careful evaluation is needed to distinguish hypersensitivity pneumonitis from disseminated infections. Unlike infection, hypersensitivity pneumonitis follows a milder, non-progressive course that improves after the discontinuation of BCG. Microbiological tests, including cultures and polymerase chain reaction (PCR) for *Mycobacterium bovis*, were consistently negative.

BAL often demonstrates lymphocytosis exceeding 50% with variable CD4/CD8 ratios. Biopsies typically reveal non-caseating granulomas with lymphocytic infiltration, supporting a hypersensitivity reaction.

Treatment involves the discontinuation of BCG and the initiation of systemic corticosteroids. Patients usually experience rapid clinical and radiological improvement within a month, confirming the non-infectious nature of hypersensitivity pneumonitis [12,13].

Acute eosinophilic pneumonia

Acute eosinophilic pneumonia is a rare but important pulmonary complication of intravesical BCG therapy for bladder carcinomas. Unlike more common reactions such as fever or cystitis, acute eosinophilic pneumonia represents a distinct hypersensitivity response rather than an infectious process. Its true incidence is unknown owing to limited reports, but it is clearly less frequent than interstitial pneumonitis or miliary tuberculosis.

Clinically, acute eosinophilic pneumonia presents with sudden-onset fever, dyspnea, nonproductive cough, and sometimes severe hypoxemic respiratory failure. Imaging typically shows diffuse alveolar or mixed alveolar-interstitial infiltrates, often described as ground-glass opacities and reticulonodular patterns. Laboratory tests usually reveal marked peripheral eosinophilia, whereas BAL often demonstrates eosinophils exceeding 25%.

Microbiological cultures and PCR for mycobacteria are essential to exclude disseminated infections, as treatment differs significantly. In the reported acute eosinophilic pneumonia cases, these tests were consistently negative, supporting a hypersensitivity mechanism. A positive lymphocyte stimulation test for BCG antigens may further confirm this diagnosis.

Management involves the immediate cessation of BCG and initiation of systemic corticosteroids. High-dose steroids typically result in rapid improvement within days. Unlike infectious complications, antituberculosis therapy is unnecessary, and recovery is usually complete without relapse after stopping steroids [11,14].

Sarcoid-like granulomatous reactions

Sarcoid-like granulomatous reactions are immune-mediated responses that cause non-caseating granulomas, typically involving mediastinal or hilar lymph nodes and occasionally the lungs. Imaging often shows lymphadenopathy with increased fluorodeoxyglucose (FDG) uptake on PET scans, closely mimicking metastatic disease or sarcoidosis and creating diagnostic uncertainty.

Definitive diagnosis requires histopathological examination. Endobronchial ultrasound-guided fine-needle aspiration usually demonstrates non-necrotizing granulomas, while microbiological tests, including acid-fast staining, cultures, and PCR, remain negative, helping exclude disseminated BCG infection.

The management is generally conservative. Most cases resolve spontaneously after discontinuing BCG therapy. Corticosteroids may be used for persistent symptoms such as cough or dyspnea, but many patients improve without medication. Antimycobacterial therapy is unnecessary in the absence of active infection [15].

Cavitary lung disease

Cavitary lung disease is a rare manifestation of BCG infection and has been described in only a few case reports. Punal et al. reported a 70-year-old man with recurrent cavitary lung lesions. Surgical pathology showed necrotizing granulomas, and cultures confirmed *Mycobacterium bovis* infection, which was attributed to prior BCG instillation. The patient was treated with antimycobacterial therapy [16].

Pleural effusion

Pleural effusion is a rare complication of intravesical BCG immunotherapy in bladder cancer. It arises when *Mycobacterium bovis* triggers either a hypersensitivity reaction or, less commonly, a direct infection of the pleural space. Patients typically present with dyspnea, fever, chest pain, and systemic symptoms that occur days to weeks after instillation.

Diagnosis requires distinguishing BCG-related effusions from tuberculosis and malignancy. Imaging usually shows unilateral or bilateral fluid collections, and pleural fluid analysis often reveals an exudate with lymphocytic predominance and elevated adenosine deaminase (ADA) levels, which can mimic tuberculous effusion. Microbiologic tests, including acid-fast staining, cultures, and PCR, are essential to exclude infection, but are frequently negative, supporting an immune-mediated process.

When diagnostic uncertainty persists, thoracoscopic, CT-guided, or ultrasound-guided pleural biopsies can help establish a diagnosis. Histopathology may reveal non-caseating granulomas consistent with a hypersensitivity reaction, whereas caseating granulomas or positive cultures indicate true infection.

Treatment typically involves discontinuing BCG therapy. Antimycobacterial treatment is reserved for cases with clear evidence of infection confirmed by biopsy or microbiology [17,18].

Diffuse alveolar damage

Diffuse alveolar damage is a rare but severe pulmonary complication associated with intravesical BCG therapy in bladder cancer. It is the histopathological correlate of acute respiratory distress syndrome (ARDS), marked by widespread alveolar injury, hyaline membrane formation, interstitial edema, and inflammation. The proposed mechanisms include hypersensitivity reactions to BCG antigens and true disseminated *Mycobacterium bovis *infection.

Clinically, patients develop rapidly worsening dyspnea, hypoxemia, fever, and diffuse pulmonary infiltrates. Imaging typically shows extensive bilateral ground-glass opacities, consolidation, and, sometimes, miliary nodularity. Bronchoscopy and microbiological tests, including culture and PCR, can help confirm infection, although the results are often negative, supporting an immune-mediated process.

Histopathology is crucial for diagnosis. Lung biopsy and autopsy revealed granulomatous inflammation and diffuse alveolar damage. Some cases show organizing diffuse alveolar damage with granulomas, suggesting a mixed mechanism, whereas others demonstrate only alveolar damage, favoring a hypersensitivity response.

Treatment involves stopping BCG instillations and starting empiric antimycobacterial therapy, typically with isoniazid, rifampicin, and ethambutol. Systemic corticosteroids are often administered, particularly when hypersensitivity or severe respiratory failure is suspected. Early steroid use has been linked to clinical improvement.

Despite therapy, prognosis can be poor, with rapid progression to respiratory failure and high mortality, particularly in frail patients. Prompt recognition and differentiation of ARDS from miliary tuberculosis, bacterial pneumonia, and other causes are essential for effective management [19-21].

Miliary tuberculosis

Miliary tuberculosis is a rare, potentially life-threatening systemic complication of intravesical BCG immunotherapy for superficial bladder cancer [3]. Disseminated infection arises when *Mycobacterium bovis* spreads hematogenously after mucosal disruption or local invasion, leading to diffuse pulmonary involvement and occasionally extrapulmonary disease.

Clinically, patients typically present with nonspecific systemic symptoms including persistent fever, fatigue, weight loss, and malaise. Respiratory manifestations such as cough or dyspnea may develop as the disease progresses. Radiologically, chest imaging often demonstrates a diffuse micronodular interstitial miliary pattern, which is most evident on high-resolution CT. These findings closely mimic those of metastatic malignancies and other granulomatous infections.

Microbiological confirmation is challenging because sputum and BAL cultures are frequently negative. However, *Mycobacterium bovis* can sometimes be identified through prolonged culture or nucleic acid amplification tests, including PCR, targeting the *Mycobacterium tuberculosis* complex. Histopathology may reveal non-caseating or caseating granulomas.

Management involves stopping BCG instillations and initiating appropriate antimycobacterial therapy, typically isoniazid, rifampicin, and ethambutol, while excluding pyrazinamide because of *Mycobacterium bovis* resistance. In severe cases, adjunctive corticosteroids can be used to control excessive inflammatory response. The prognosis varies depending on the timeliness of diagnosis and treatment, patient age, and comorbidities. Mortality has been reported to underscore the importance of early recognition [22-24].

In select cases of pulmonary complications after BCG therapy, observation without immediate antimicrobial treatment may be appropriate in carefully selected patients without systemic symptoms such as fever, hypoxia, or progressive pulmonary complaints, suggesting worsening infection. Some patients with diffuse nodules showed spontaneous improvement, likely reflecting a hypersensitivity response. This strategy requires shared decision-making and close monitoring to ensure early intervention if deterioration occurs. Observations are best reserved for individuals without known immunodeficiency, without significant multimorbidity likely to impair host defenses, and where reliable follow-up is feasible. This approach has been described only in individual case reports. One report described a patient who developed granulomatous pneumonitis with fever, malaise, and diffuse pulmonary nodules, and a shared decision was made to not start antimycobacterial therapy. Similar to our case, the patient demonstrated a significant spontaneous improvement over time [25].

## Conclusions

This case highlights the diagnostic challenges posed by miliary pulmonary nodules after intravesical BCG immunotherapy. Although disseminated BCG infection is a recognized and potentially life-threatening complication that requires prompt treatment, not all radiographic findings reflect an active infection. Careful observation and close follow-up may be appropriate in selected clinically stable patients without systemic deterioration or immunosuppression, as spontaneous resolution can occur.

Our experience emphasizes that management decisions should be individualized based on patient stability, the absence of progressive symptoms (such as persistent fever, hypoxia, or severe respiratory compromise), negative microbiological investigations, and the ability to ensure reliable monitoring. In such cases, observation without immediate antimycobacterial therapy is a reasonable approach.

Due to the rarity of this condition, there are no consensus guidelines defining the optimal management of pulmonary complications following BCG instillation. Further studies are needed to establish a clearer framework regarding the initiation of antimycobacterial treatment and when observation alone is appropriate. This underscores the importance of integrating clinical assessments, microbiological testing, and radiological evaluations to guide treatment decisions, avoid unnecessary therapy, and ensure patient safety.
